# The relevance of cell type- and tumor zone-specific VEGFR-2 activation in locally advanced colon cancer

**DOI:** 10.1186/s13046-015-0162-5

**Published:** 2015-05-13

**Authors:** Caren Jayasinghe, Nektaria Simiantonaki, Sylvia Habedank, Charles James Kirkpatrick

**Affiliations:** Institute of Pathology, University Medical Center, Johannes Gutenberg University, Langenbeckstr. 1, 55101 Mainz, Germany; Department of Pathology, Laboratory Dr. Wisplinghoff, Geibelstr. 2, 50931 Cologne, Germany; Laboratory Habedank, 12281 Berlin, Germany

**Keywords:** Colon cancer metastasis, VEGF-C, VEGF-D, VEGFR-2, pVEGFR-2

## Abstract

**Background:**

For the successful therapeutic use of inhibitors of the vascular endothelial growth factor receptor (VEGFR) pathway detailed knowledge of the mechanisms leading to tumor progression is indispensable. The main goal of this study was to determine the relevance of the VEGFR-2 activating pathway for colon carcinoma (CC) metastasis. The initial event is ligand-induced receptor activation through tyrosine autophosphorylation.

**Methods:**

VEGFR-2, its ligands VEGF-C and VEGF-D and the phosphorylated (activated) receptor forms pVEGFR-2^Tyr1175^ and pVEGFR-2^Tyr1214^ were investigated immunohistochemically in different tumor compartments (intratumoral (zone 1) - invasive front (zone 2) – extratumoral soft tissue (zone 3)) and various cell types (tumor cells, inflammatory cells, macro- and microvasculature) in 84 non-metastatic, lymphogenous-metastatic and haematogenous-metastatic CC.

**Results:**

VEGF-D produced by tumor cells has an autocrine affinity for its receptor VEGFR-2. In tumor budding regions VEGF-D-induced receptor activation by autophosphorylation at Tyr1214 seems to be a possible initial event of the VEGFR-2-mediated signaling pathway, but without effect on metastatic behaviour. In inflammatory cells of almost all CC VEGFR-2 phosphorylation at Tyr 1175 and Tyr 1214 was detectable without accompanying receptor expression, suggesting receptor activation without cell surface expression. Peritumoral inflammatory cells also expressed paracrine acting VEGF-C. The autocrine VEGF-D/VEGFR-2 signaling axis and receptor autophosphorylation at Tyr1214 appear to be main events for capillaries in all three tumor zones and for small vessels in zone 1 and 2. Independent of the metastatic status a large number of cases with capillary immunopositivity in the angiogenically active invasive front was documented, especially for VEGF-D, VEGFR-2 and pVEGFR-2^Tyr1214^. VEGFR-2 positive extratumoral capillaries were significantly more common in distant metastatic CC. In all tumor compartments the investigated biomolecules were also detected in different frequencies in the macrovasculature, which is responsible for sufficient tumor vascularization. In addition, vascular paracrine-acting VEGF-C production was widely detected, but without zone and vessel-type dependence.

**Conclusions:**

The VEGFR-2 activating pathway is closely involved in tumor cell-associated, vessel-mediated and immuno-inflammatory processes in colon carcinoma and appears to contribute to tumor survival and growth as well as maintenance of the infiltrative phenotype rather than to promote metastasis.

## Background

More than ten years ago targeted therapy was heralded as the beginning of a new era in anti-cancer therapy. The goal of targeted therapy is to interfere with specific molecules in the tumor to block its growth and spread [1]. Angiogenesis plays a central role in the processes of tumor proliferation and metastasis [2]. In angiogenic signaling, vascular endothelial growth factor A (VEGF-A, also referred to as VEGF) is the most potent angiogenic factor and its receptor, VEGFR-2, is the predominant mediator. Thus it is not surprising, that right from the beginning especially VEGF and VEGFR-2 have been the main targets for antiangiogenic therapy [3]. However, the efficacy of antiangiogenic agents appears to be limited to a subset of the patient cohort. A challenge at the present time is the establishment of criteria for selecting the patient group that would benefit from such targeted therapy. This presumes, however, detailed knowledge of the specific expression patterns of the target molecules and their interactive partners within the tumor tissue, e.g. the presence of the biomolecule, cell-type-affinity, tumor compartment distribution and activity status. Potential intratumoral and intertumoral differences in the expression of these factors in various stages of tumor progression can be better assessed *in situ*. Indeed, in histological specimens a sophisticated examination of the behavior pattern of tumor cells themselves and components of the tumor microenvironment in the different tumor zones is possible and crucial, because firstly, tumor tissue is highly heterogeneous, and secondly, angiogenesis, tumor progression and metastasis are decisively regulated by tumor-host interactions. Finally, functional compartmentalization and topological organization are essential for tumor survival and progression [4–6].

The present study focuses on the expression profiles of members of the VEGFR-2 – activating pathway in colon cancer (CC) tumor tissue. Previously, in an immunohistochemical analysis of the VEGFR-1 – activating pathway in CC, we have shown that VEGF is abundantly expressed by tumor cells as well as micro- and macrovasculature, but without significant correlation with lymphogenous or haematogenous metastasis [7]. After its binding to VEGF, VEGFR-2 dimerizes and undergoes autophosphorylation of tyrosine residues within its cytoplasmic domain, thus initiating the downstream signaling cascades [8]. Takahashi et al. identified Tyr1175 and Tyr1214 as major autophosphorylation sites in VEGFR-2, which are located in the C-terminal domain [9]. Tyr1175 is essential for proliferation and migration of endothelial cells, development of endothelial cells from progenitor cells, vascular secretion of von Willebrand factor and the process of vasculogenesis [8]. The Tyr1214 residue is required to trigger VEGF-induced actin remodelling and endothelial migration [10]. Data on the phosphorylated (activated) form of VEGFR-2 in tumor tissue and its relevance for tumor progression are very sparse until now. In addition to VEGF, VEGFR-2 binds proteolytically processed VEGF-C and VEGF-D [11]. VEGF-C stimulates receptor dimerization, leading to the formation of VEGFR-2/VEGFR-3 heterodimers and VEGFR-3/VEGFR-3 homodimers, which are implicated in angiogenesis and lymphangiogenesis, respectively [12]. Moreover, VEGF-D activates both receptors, VEGFR-2 and VEGFR-3, and drives angiogenesis and lymphangiogenesis [13]. In animal models of cancer, expression of VEGF-C and VEGF-D consistently promotes growth of blood vessels and lymphatics in and around tumors, thus facilitating tumor growth and enhancing lymph node and distant organ metastasis. However, the clinical significance of the VEGF-C and VEGF-D/VEGFRs axes for tumor progression is unclear. With respect to the involvement of VEGF-C and VEGF-D in colorectal cancer metastasis there are conflicting reports [14].

In order to determine the relevance of the VEGFR-2 activating pathway for CC metastasis we investigated the protein expression profiles of the total and phosphorylated forms (Tyr1175 and Tyr1214) of this receptor and its ligands VEGF-C and VEGF-D in tumor cells as well as the main components of the tumor microenvironment, namely tumor-associated vasculature and inflammatory response in non-metastatic, lymphogenous- and haematogenous-metastatic sporadic CC. Taking tumor heterogeneity into consideration, the tumor tissue was subdivided in three separately investigated, strategically important compartments, in particular, tumor center (zone 1), invasive margin (zone 2) and tumor-free surrounding soft tissue (zone 3).

## Material and methods

### Ethics statement

Ethical approval was granted by the Clinical Research Ethics Commitee of the federal state of Rhineland-Palatinate (Mainz, Germany). Written informed consent was obtained from all patients.

### Tissue samples

The CC tissue samples used in this study derived from 84 patients with an average age of 65.2 (range 52–83) undergoing elective surgery for sporadic CC at the University of Mainz during the years 1998–2003. All tumors were staged following the guidelines of the TNM Classification of Malignant Tumors. With respect to the T status all tumors investigated were T3 (infiltration of subserosa) and moderately differentiated. According to metastatic status 36 of them were non-metastatic (N0/M0), 24 lymphogenous-metastatic (N+) and 24 haematogenous-metastatic (M+) CC at the time of diagnosis.

### Immunohistochemistry

All immunohistochemical reactions were conducted on formalin-fixed and paraffin-embedded samples.

*VEGF-C, VEGF-D VEGFR-2, pVEGFR-2*^*Tyr1175*^*and pVEGFR-2*^*Tyr1214*^: After deparaffination endogenous peroxidase activity was blocked with hydrogen peroxide. Heat-induced epitope retrieval was performed in citrate buffer pH 6,0 for 8 min. using a pressure cooker. The detection kits ZytoChem Plus HRP Kit, anti-Rabbit and ZytoChem Plus (HRP) Polymer Kit, anti-Mouse (Zytomed Systems, Berlin, Germany) were utilized following the manufacturer’s instructions. The primary antibodies were applied for 45 min. at room temperature and diluted as follows: rabbit polyclonal anti-VEGF-C (Abcam, Cambridge, UK) 1:800, rabbit polyclonal anti-VEGF-D (Abcam) 1:200, rabbit monoclonal anti-VEGFR-2 (SP123, Abcam) 1:1000, rabbit polyclonal anti-phosphoVEGFR-2 (pY1175, Abcam), 1:100 and rabbit polyclonal Anti-phosphoVEGFR-2 (pY1214, Abcam) 1:100. Staining was completed with Novolink Max DAB (Polymer) Kit (Leica Biosystems, Wetzlar, Germany).

Sections were counterstained with Mayer’s hematoxylin (Thermo Fisher Scientific, Fremont, USA). To prove the specificity of the immunoreactions, CC samples were stained solely with the secondary antibody, omitting the primary antibody, and served as negative control.

Immunostaining reactions of each sample were evaluated independently by two authors (CJ and NS) without knowledge of the metastatic status. The endothelial and inflammatory cell staining was judged as either negative or positive. The intensity of the tumoral staining was scored on a semiquantitative scale from 0 to 2 (0: no staining, 1: weak staining, 2: strong staining). In most cases the staining was homogeneous. In those cases where heterogeneous staining was observed, that level of staining intensity which was visible in more than 50 % of the cells was chosen for the classification into a defined group. Tumor budding was defined as single tumor cells and oligocellular tumor cell clusters (≤5 cells) along the tumor invasion front. It was delineated as low (< 1/3 of invasion front) and high (>1/3 of invasion front).

### Statistical analysis

Statistical significance was assessed using Fisher’s exact test. p < 0.05 was considered to be statistically significant. The correlations between expression of VEGFR-2 and its ligands as well as pVEGFR-2 were assessed with the Spearman’s rank test.

## Results

### Tumor cell- associated VEGFR-2 activation in CC tissue

VEGF-C and VEGF-D were expressed in the cytoplasm of tumor cells by 65 % and 52 % of the CC, respectively (Table 1). In tumor budding regions, which reflect the spreading capacity of tumor cells, the percentage distribution of cases with positive VEGFR-2 ligand immunoreactivity was similar to the tumor center, namely 71 % for VEGF-C and 55 % for VEGF-D (Table 1). Both ligands were detected with uniform staining intensity and distribution within the three compared tumor fractions. There were no significant associations between metastasis-free and metastatic carcinomas. Ligand/VEGFR-2 co-expression profiles revealed a nearly uniform distribution for VEGF-D/VEGFR-2 and an accentuated co-reactivity in the tumor budding regions for VEGF-C/VEGFR-2 in the respective metastatic group (Table 2). However, there were no significant differences concerning the metastatic state. Correlation analysis displayed a VEGF-D/VEGFR-2 ligand-receptor affinity in tumor cells located in the tumor center and in tumor budding regions (r = 0.425, p = 0.0001 in tumor center and r = 0.421 and p = 0.0002 in tumor budding; Table 3).

**Table 1:** Percentage distribution of the VEGFR-2 ligands VEGF-C and VEGF-D in tumor cells of CC tissue

	Score	N0/M0	N+	M+	CC
		%	%	%	%
*Tumor center*					
	0	37	33	34	35
VEGF-C	1	34	42	57	65
	2	29	25	9	
	0	39	50	54	48
VEGF-D	1	50	50	33	52
	2	11	0	13	
*Tumor budding*					
	0	35	35	16	29
VEGF-C	1	35	40	74	71
	2	30	25	10	
	0	33	50	52	45
VEGF-D	1	63	50	39	55
	2	4	0	9	

The intensity of the tumoral staining was scored on a semiquantitative scale from 0 to 2 for the investigated biomolecule (0: no staining, 1: weak staining, 2: strong staining). For the statistical analysis using Fisher’s exact test the examined cases were separated into two groups, characterized by a negative/positive expression for VEGF-C and VEGF-D. The line in the staining intensity column indicates this dichotomization for each biomolecule. There were no significant differences between the groups

**Table 2:** Numerical and percentage distribution of ligand/VEGFR-2 and VEGFR-2/pVEGFR-2 co-expression in the tumor cells

	N0/M0	N+	M+
	n (%)	n (%)	n (%)
*Tumor center*			
VEGF-C+/VEGFR-2+	22/14 (64)	16/9 (56)	14/7 (50)
VEGF-D+/VEGFR-2+	22/16 (73)	12/10 (83)	11/7 (64)
VEGFR-2+/pVEGFR-2^Tyr1175^+	19/14 (74)	13/11 (85)	12/5 (42)
VEGFR-2+/pVEGFR-2^Tyr1214^+	19/17 (89)	13/10 (77)	13/11 (85)
*Tumor budding*			
VEGF-C+/VEGFR-2+	16/12 (75)	10/7 (70)	10/8 (80)
VEGF-D+/ VEGFR-2+	16/13 (81)	10/8 (80)	13/8 (62)
VEGFR-2+/pVEGFR-2^Tyr1175^+	16/11 (69)	10/8 (80)	12/7 (58)
VEGFR-2+/pVEGFR-2^Tyr1214^+	16/16 (100)	10/9 (90)	13/13 (100)

n: total number of ligand positive cases/total number of ligand positive cases with concomitant VEGFR-2 positivity or total number of VEGFR-2 positive cases/total number of VEGFR-2 positive cases with concomitant pVEGFR-2 positivity. There were no significant differences between the groups

**Table 3:** Numerical distribution and statistical significance of ligand/VEGFR-2 correlations in tumor cells of CC tissue

VEGFR-2	VEGF-C		p-value	VEGF-D		p-value
	+	-	(r)	+	-	(r)
*Tumor center*						
+	30	22	NS	33	12	0.0001
-	11	16		12	27	(0.425)
*Tumor budding*						
+	27	9	NS	29	10	0.0002
-	20	10		10	21	(0.421)

Positive tumoral expression of VEGFR-2 is positively correlated with positive tumoral VEGF-D expression in tumor center and tumor budding regions. r = Spearman rank correlation coefficient. p < 0.05 was taken as statistically significant. NS, not significant

**Fig. 1:**
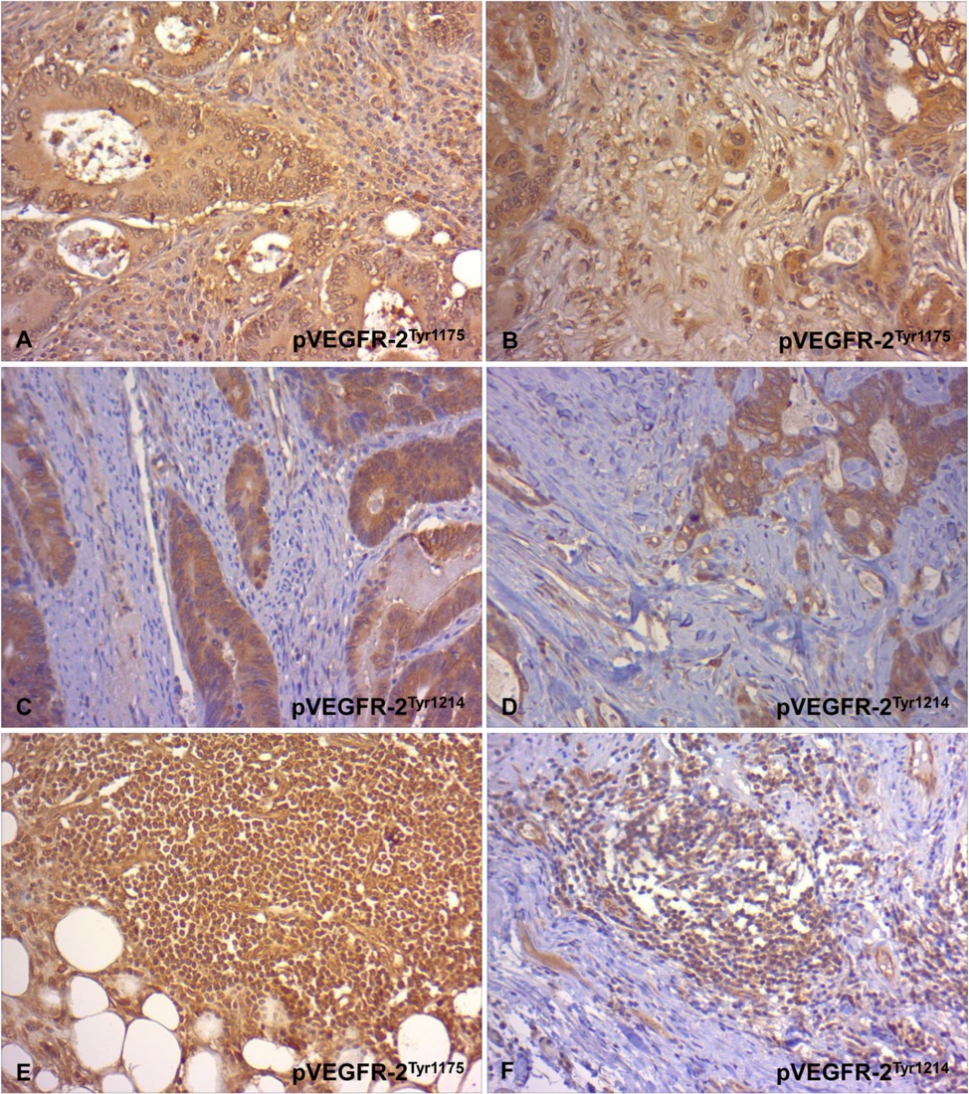
Immunohistochemical staining of pVEGFR-2 in tumor cells and inflammatory cells of CC tissue. **a**,**b** Characteristic pVEGFR-2^Tyr1175^ expression in tumor cells with submembranous and nuclear immunostaining in the tumor center (**a**, x 200) and tumor budding regions (**b**, x 200). **c**,**d** Characteristic pVEGFR-2^Tyr1214^ expression in tumor cells with submembranous and nuclear immunostaining in the tumor center (**c**, x 200) and tumor budding regions (**d**, x 200). **e** Characteristic VEGFR-2^Ty1175^ expression in inflammatory cells (x 200). **f** Characteristic VEGFR-2^Ty1214^ expression in inflammatory cells (x 200)

In the tumor center and tumor budding regions 54 and 55 % of the CC, respectively, showed a positive cytoplasmic VEGFR-2 immunoreaction in tumor cells without significant differences among the comparative groups (Table 4). Positive tumoral staining for pVEGFR-2^Tyr1175^ and pVEGFR-2^Tyr11214^ was observed in 64 and 80 % in the tumor core and 67 and 89 % in tumor budding regions, respectively, but without significant correlation with metastasis (Table 4). pVEGFR-2^Tyr1175^ expression in tumor cells had a detectable submembranously accentuated cytoplasmic, and, additionally, nuclear staining in virtually all positive CC (Fig. 1a and b). pVEGFR-2^Tyr1214^ was seen consistently in tumor cells of most CC, but with differing cytoplasmic immunostaining intensity (Table 4). In addition to a submembranously accentuated cytoplasmic staining a nuclear immunoreactivity was seen in 64 % of the cases (Table 4 and Fig. 1c and d). Neither the cytoplasmic nor the nuclear expression in tumor cells was statistically significant with respect to metastasis. However, significant correlations were found between VEGFR-2 and pVEGFR-2^Tyr1214^ expression in tumor budding regions (r = 0.286, p = 0.017; Table 5). Since a concomitant VEGFR-2/pVEGFR-2 immunopositivity can be interpreted as a potentially ligand-dependent tyrosine autophosphorylation, co-expression profiles were analyzed as well. VEGFR-2^Tyr1175^ co-expression was observed most frequently in N+ CC and most rarely in M+ CC (Table 2), although significant differences were not established. Interestingly, almost all tumoral VEGFR-2 positive cases exhibited a concomitant pVEGFR-2^Tyr1214^ expression in tumor budding regions.

**Table 4:** Percentage distribution and statistical significance of VEGFR-2 and pVEGFR-2 in tumor cells of CC tissue

	Score	N0/M0	N+	M+	CC
		%	%	%	%
*Tumor center*					
VEGFR-2	0	47	46	46	46
	1	39	46	50	54
	2	14	8	4	
Cytoplasmatic VEGFR-2^Tyr1175^	0	36	29	43	36
	1	14	17	14	64
	2	50	54	43	
Nuclear VEGFR-2^Tyr1175^	-	6	4	4	5
	+	94	96	96	95
Cytoplasmatic VEGFR-2^Tyr1214^	0	26	26	8	20
	1	31	26	29	80
	2	43	48	63	
Nuclear VEGFR-2^Tyr1214^	-	31	26	50	36
	+	69	74	50	64
*Tumor budding*					
VEGFR-2	0	41	50	43	45
	1	48	10	43	55
	2	11	40	14	
Cytoplasmatic VEGFR-2^Tyr1175^	0	33	30	36	33
	1	15	5	14	67
	2	52	65	50	
Nuclear VEGFR-2^Tyr1175^	-	4	5	0	3
	+	96	95	100	97
Cytoplasmatic VEGFR-2^Tyr1214^	0	11	21	0	11
	1	22	26	17	89
	2	67	53	83	
Nuclear VEGFR-2^Tyr1214^	-	36	21	61	38
	+	67	79	39	62

The intensity of the tumoral staining was scored on a semiquantitative scale from 0 to 2 for the investigated biomolecule (0: no staining, 1: weak staining, 2: strong staining). For the statistical analysis using Fisher’s exact test the examined cases were separated into two groups characterized by a negative/positive expression for VEGFR-2, VEGFR-2^Tyr1175^ and VEGFR-2^Tyr1214^. The line in the staining intensity column indicates this dichotomization for each biomolecule. There were no significant differences between the groups

**Table 5:** Numerical distribution and statistical significance of VEGFR-2/pVEGFR-2 correlations in tumor cells of CC tissue

VEGFR-2	pVEGFR-2^Tyr1175^		p-value	pVEGFR-2^Tyr1214^		p-value
	+	−	(r)	+	−	(r)
*Tumor center*						
+	30	14	NS	38	7	NS
-	23	16		27	10	
*Tumor budding*						
+	26	12	NS	38	1	0.017
-	20	11		24	6	(0.286)

Positive tumoral expression of VEGFR-2 is positively correlated with positive tumoral VEGFR-2^Tyr1214^ expression in tumor budding regions. r = Spearman rank correlation coefficient. p < 0.05 was taken as statistically significant. NS, not significant

### Inflammatory cell-associated VEGFR-2 activation in CC tissue

VEGF-C was markedly expressed in inflammatory cells in 80–94 % of the cases, independent of the tumor zone (Table 6). VEGF-D expression was sporadic and occurred in 6 and 5 %, respectively, of N0/M0 and N+ carcinomas in the tumor center and in 14 and 27 %, respectively, along the invasive front. None of the M+ CC showed VEGF-D immunopositivity. Only few non-metastatic CC in zone 1 and 2 and merely two N+ cases in zone 2 displayed a tumor-associated VEGFR-2 positive inflammatory reaction. In contrast, independent of the metastatic status and tumor compartment both phosphorylated VEGFR-2 forms showed a positive immunoreaction in virtually all carcinomas (Table 6 and Fig. 1e and f).

**Table 6:** Percentage distribution of the VEGF-C, VEGF-D, VEGFR-2 and pVEGFR-2 in inflammatory cells of CC tissue

	N0/M0 (%)		N+ (%)		M+ (%)	
	zone1	zone2	zone1	zone2	zone1	zone2
VEGF-C	88	80	94	86	88	90
VEGF-D	6	14	5	27	0	0
VEGFR-2	12	22	0	9	0	0
VEGFR-2^Tyr1175^	94	97	94	100	90	100
pVEGFR-2^Tyr1214^	90	100	94	100	95	100

There were no significant differences between the groups

### Vasculature-associated VEGFR-2 activation in CC tissue

The vascular expression profiles of the VEGFR-2 activating pathway were investigated separately in three vessel types (large vessels, small vessels and capillaries) within the three zones.

**Fig. 2:**
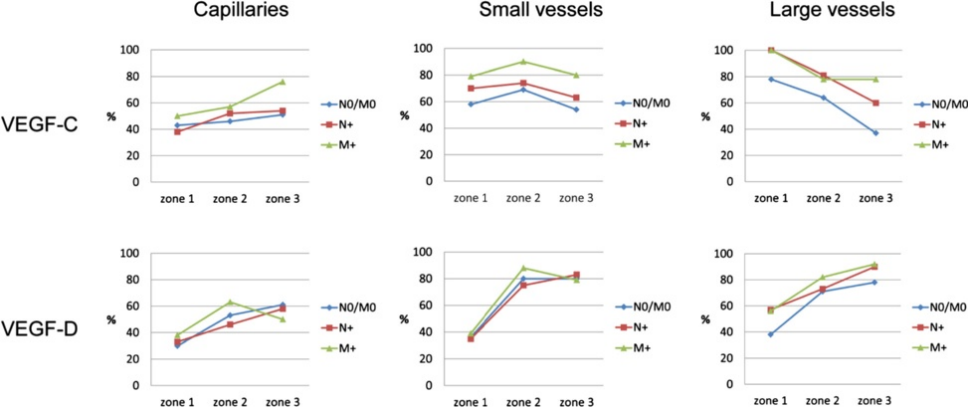
Graphical presentation of percentage distribution of VEGF-C and VEGF-D, in the vasculature of CC tissue

In about half of the investigated carcinomas there was a positive endothelial VEGF-C expression in capillaries, with a slight increase in the number of positive cases from the tumor center towards the invasive front and surrounding extratumoral tissue (Fig. 2). Particularly noteworthy is the large number of cases with VEGF-C-positive macrovascular vessels in all tumor compartments, especially large vessels in the tumor core of metastasizing carcinomas. However, no significant differences could be established with respect to the metastatic status.

**Fig. 3:**
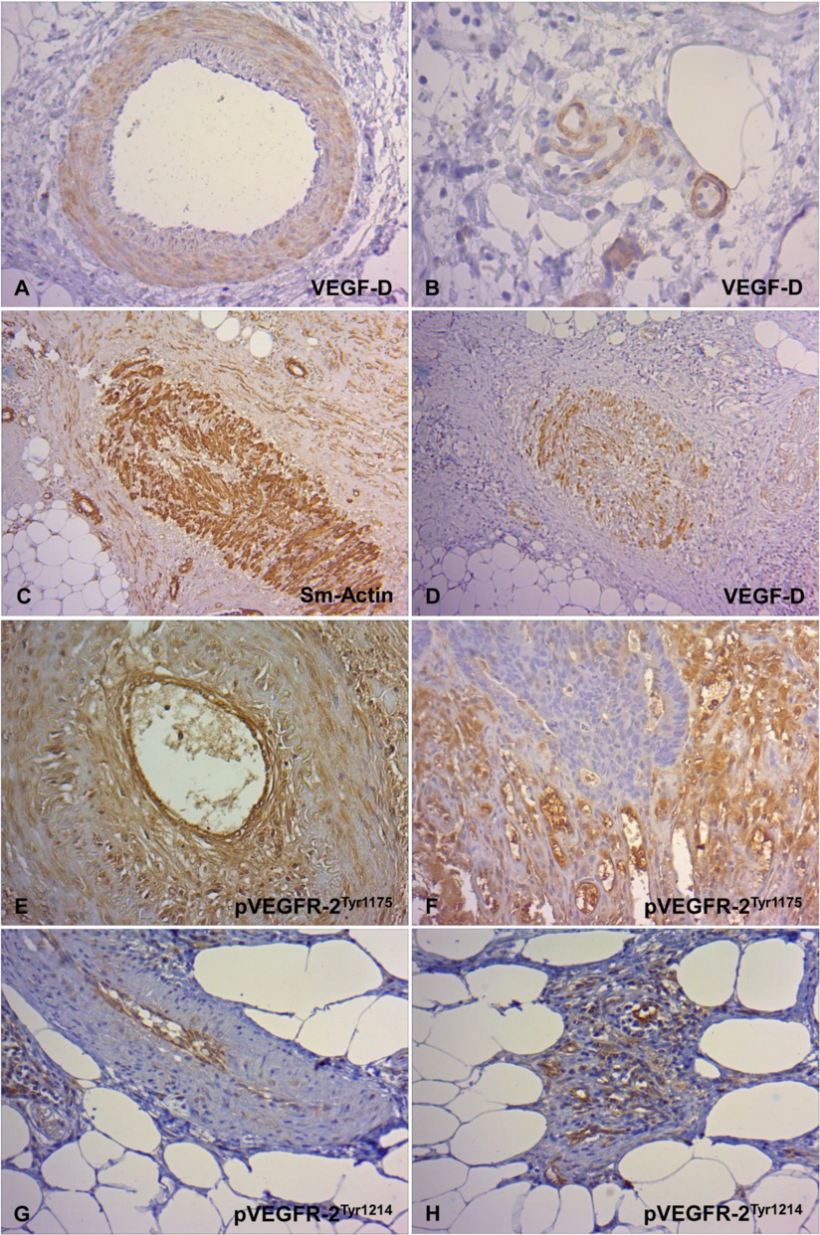
Immunohistochemical staining of the VEGF-D and pVEGFR-1 in the vasculature of CC tissue. **a**,**b** Characteristic vascular VEGF-D expression. VEGF-D-positive macrovascular vessels with immunoreactivity in smooth muscle cells of the media layer and occasionally in endothelial cells (**a**, x 200) and VEGF-D-positive capillaries with endothelial immunoreactivity (**b**, x 400). Altered macrovascular vessels with discontinuous, hypoplastic smooth muscle cell layer (**c**, Sm-Actin., x 100) and VEGF-D immunopositivity (**d**, x 100). **e**,**f** Characteristic endothelial VEGFR-2^Tyr1175^ expression in macrovessels (**e**, x 200) and capillaries (**f**, x200). **g**,**h** Characteristic endothelial VEGFR-2^Tyr1214^ expression in macrovessels (**g**, x 200) and capillaries (**h**, x 200)

Independent of the tumor stage, about 40 % of the carcinomas had a positive capillary and small vessel-related VEGF-D immunoreactivity and 40–60 % had a large vessel-related VEGF-D immunopositivity in the tumor center (Fig. 2). A marked increase of cases with VEGF-D positive capillaries and especially macrovascular vessels from zone 1 to zone 2 and zone 3 was observed. Interestingly, expression of VEGF-D in the macrovasculature was mainly located in the smooth muscle cells of the media layer and occasionally in endothelial cells (Fig. 3a). In the capillaries VEGF-D was expressed by endothelial cells and pericytes (Fig. 3b). Immature blood vessels with discontinuously hypoplastic muscle wall layers were also stained (Fig. 3c,d).

**Fig. 4:**
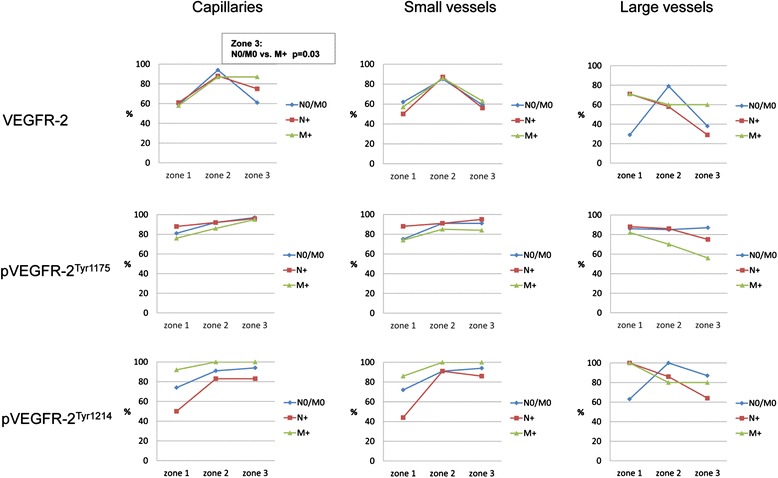
Graphical presentation of percentage distribution of VEGFR-2, and pVEGFR-2 in the vasculature of CC tissue

A zonally subdivided endothelial VEGFR-2 expression in 58, 90 and 75 % of the cases for capillaries and 56, 86 and 60 % for small vessels was observed with a remarkably uniform distribution among the investigated groups (Fig. 4). A pronounced increase of cases with VEGFR-2 positivity in both vascular types was documented from zone 1 to zone 2. In the invasive front 90 % of the vessels showed a positive reaction. In zone 3 there were significantly more cases with VEGFR-2 positive capillaries in the distant-metastasic situation in comparison to the non-metastatic stage (p = 0.03). In all groups large vessel-associated positive VEGFR-2 immunoreactivity was often present along the invasive front and additionally in the tumor center in metastatic carcinomas (N+ and M+) as well as extratumorally in distant-metastatic CC.

Regardless of the metastatic state, the tumor tissue exhibited endothelial expression of pVEGFR-2^Tyr1175^ in all segments of the vascular system and was uniform in all zones in a large number of cases (Figs. 3e,f and 4). Endothelial expression of pVEGFR-2^Tyr1214^ was detectable in the macro- and microvasculature in a large number of cases, with a different zonal distribution pattern (Figs. 3g,h and 4). From zone 1 to zone 2 a marked increase in cases with positive capillaries and small vessels was observed in the non-metastatic and lymphogenously-metastatic CC. In contrast, in distant-metastatic cases these vessel types showed an approximately constant percentage distribution in all three zones. Large vessel-associated positive pVEGFR-2^Tyr1214^ immunoreactivity was consistently present in all cases and in all tumor compartments.

Ligand/VEGFR-2 co-expression analysis indicated in zone 2 a co-expression of VEGF-C/D and VEGFR-2 in capillaries in almost all cases and in small vessels in about 90 % of the cases (Table 7). In 83–100 % of the cases a capillary and in 77–100 % of the cases a small vessel-associated combined VEGFR-2/pVEGFR-1Tyr^1175^ expression was demonstrated, which was zone-dependent. 88 % of the nodal-positive cases displayed a concomitant VEGFR-2/pVEGFR-2^Tyr1214^ expression in capillaries and small vessels (Table 7). In the other groups an endothelial positivity for the VEGFR-2 total protein was almost always accompanied by a simultaneous pVEGFR-2Tyr^1214^ positivity (91-100 %), independent of the metastatic status, tumor zone and vessel type.

**Table 7:** Numerical and percentage distribution of ligand/VEGFR-2 and VEGFR-2/pVEGFR-2 co-expression in the vasculature

		N0/M0	N+	M+
		n (%)	n (%)	n (%)
*VEGF-C+/VEGFR-2+*				
Capillaries	zone 1	20/8 (40)	9/6 (67)	9/6 (67)
	zone 2	16/16 (100)	11/11 (100)	11/9 (82)
	zone 3	18/11 (61)	13/11 (85)	16/14 (88)
Small vessels	zone 1	16/12 (75)	11/6 (55)	15/8 (53)
	zone 2	23/21 (91)	16/15 (94)	17/14 (82)
	zone 3	17/9 (53)	14/8 (57)	13/7 (54)
*VEGF-D+/VEGFR-2+*				
Capillaries	zone 1	11/10 (91)	8/7 (88)	9/8 (89)
	zone 2	19/19 (100)	11/11 (100)	15/14 (93)
	zone 3	21/15 (71)	14/12 (86)	12/12 (100)
Small vessels	zone 1	10/9 (90)	6/4 (67)	8/5 (63)
	zone 2	27/24 (89)	17/16 (94)	19/17 (89)
	zone 3	26/16 (62)	19/11 (58)	15/10 (67)
*VEGFR-2+/VEGFR-2* ^*Tyr1175*^ *+*				
Capillaries	zone 1	20/17 (85)	14/14 (100)	12/10 (83)
	zone 2	34/31 (91)	21/19 (90)	18/15 (83)
	zone 3	22/22 (100)	18/17 (94)	19/18 (95)
Small vessels	zone 1	13/10 (77)	8/8 (100)	11/9 (82)
	zone 2	29/26 (90)	20/18 (90)	16/14 (88)
	zone 3	18/16 (89)	12/12 (100)	9/8 (89)
*VEGFR-2+/VEGFR-2* ^*Tyr1214*^ *+*				
Capillaries	zone 1	19/18 (95)	13/10 (77)	14/13 (93)
	zone 2	33/32 (97)	21/19 (90)	21/21 (100)
	zone 3	21/21 (100)	15/15 (100)	21/21 (100)
Small vessels	zone 1	15/14 (93)	8/6 (75)	11/10 (91)
	zone 2	27/27 (100)	20/19 (95)	19/19 (100)
	zone 3	17/17 (100)	12/11 (92)	12/12 (100)

n: total number of ligand positive cases / total number of ligand positive cases with concomitant VEGFR-2 positivity or total number of VEGFR-2 positive cases / total number of VEGFR-2 positive cases with concomitant pVEGFR-2 positivity. There were no significant differences between the groups

Significant correlations were found between VEGFR-2 and VEGF-D in vascular expression, except for the small vessels in zone 3 (Table 8). A similar pattern was noted in the comparison between VEGFR-2 and pVEGFR-2^Tyr1214^ expression (Table 8). Additionally, a significant association between VEGFR-2 and pVEGFR-2^Tyr1175^ in intratumorally located capillaries was observed (r = 0.234, p = 0.036; data not shown).

**Table 8:** Numerical distribution and statistical significance of VEGF-D/VEGFR-2 and VEGFR-2/pVEGFR-2^Tyr1214^ correlations in the vasculature of CC tissue

VEGFR-2	VEGF-D		p-value	VEGFR-2^Tyr1214^		p-value
+/−	+	-	(r)	+	-	(r)
Ca zone 1	25	3	0.0001	41	5	0.0001
	23	32	(0.454)	18	16	(0.406)
Ca zone 2	44	1	0.014	72	3	0.0001
	32	7	(0.267)	3	4	(0.531)
Ca zone 3	39	8	0.025	57	1	0.001
	21	14	(0.256)	17	5	(0.356)
SV zone 1	18	6	0.013	40	4	0.0001
	17	20	(0.313)	12	14	(0.494)
SV zone 2	57	6	0.034	65	1	0.0001
	11	5	(0.236)	6	4	(0.524)
SV zone 3	37	23	NS	40	1	NS
	6	7		25	4	

Positive vascular expression of VEGFR-2 is positively correlated with positive vascular VEGF-D expression and positive vascular VEGFR-2^Tyr1214^ expression in capillaries and small vessels in zone 1 and 2 and capillaries in zone 3. r = Spearman rank correlation coefficient. p < 0.05 was taken as statistically significant. NS, not significant. Ca = capillaries; SV = small vessels

**Fig. 5:**
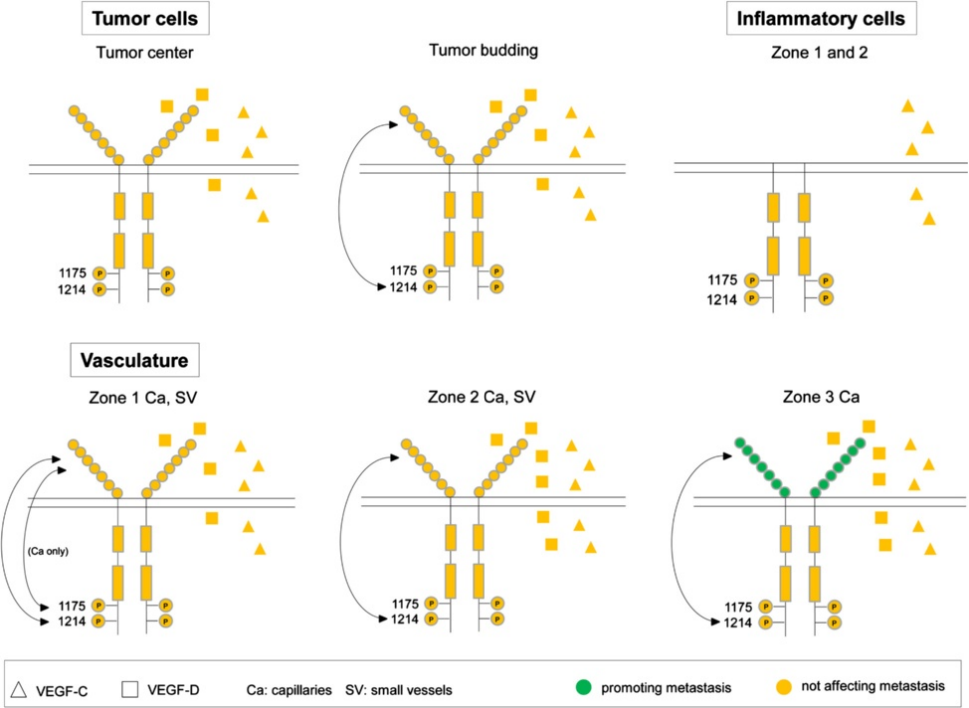
Schematic presentation of VEGFR-2 activation in CC tissue and its association with metastasis. VEGF-D produced by tumor cells in the tumor center and in tumor budding regions has an autocrine affinity for its receptor VEGFR-2. In dissociated tumor cells VEGF-D-mediated receptor activation by autophosphorylation at Tyr1214 seems to be a potential signaling pathway but without effect on the metastatic potential. Tumor cells produce paracrine-acting VEGF-C as well. In inflammatory cells of almost all colon carcinomas there is VEGFR-2 phosphorylation at Tyr 1175 and Tyr 1214 in the tumor center (zone 1) and invasive front (zone 2), without accompanying receptor expression, suggesting receptor activation without cell surface expression. Inflammatory cells are also a possible source of paracrine-acting VEGF-C. Autocrine VEGF-D/VEGFR-2 signaling axis and receptor autophosphorylation at Tyr1214 seem to be main events in CC for capillaries in all three tumor zones and for small vessels in zone 1 and 2. Additionally, the VEGFR-2 receptor of intratumoral microvessels has a close association with its cytoplasmic tyrosine residue Tyr 1175. Independent of the metastatic status an increase of the number of cases can be demonstrated with capillary immunopositivity especially for VEGF-D, VEGFR-2 and pVEGFR-2^Tyr1214^ in the angiogenically active invasive front. Remarkably, these biomolecules were also often detected in small vessels of marginal tumor areas (zone 2) which are responsible for sufficient tumor vascularization. VEGFR-2 expression in extratumoral capillaries (zone 3) was significantly more common in distant metastatic CC. In addition, paracrine-acting VEGF-C production was independent of the zone and vessel type

As there were only a small number of cases with detectable large vessels, this vessel type was not considered for statistical evaluation of co-expression and correlation analyses. Fig. 5 represents a summary of our results in a schematic form.

Notably, tumoral and vascular correlation analyses with previously published expression profiles of the other VEGFR-2 ligand, VEGF-A, which were investigated in the same tumor specimens as in the present study, did not lead to any significant results [7]. Additionally, the VEGF-A/VEGFR-2 co-expression analysis revealed no statistically significant differences regarding the metastatic stage.

In the present study expression analysis in lymphatic vessels was not performed. Based on our experience with colon carcinoma tissue, a detailed and reproducible examination of biomolecules localized exclusively in lymphatic vessels is not possible with conventional immunohistochemistry.

## Discussion

The present study centers on three strategically important compartments of colon carcinoma (CC) tissue (tumor center - invasive front - adjacent tumor-free soft tissue) and represents a systematic analysis of the expression of total and phosphorylated VEGFR-2 and its ligands VEGF-C and VEGF-D in the vasculature, tumor cells and inflammatory cells in relation to metastatic status.

We demonstrated that CC are characterized by common immunopositivity for VEGF-C in tumor cells as well as the macro- and microvasculature and peritumoral inflammation. Nevertheless, VEGF-C was not found to be linked to the metastatic status. Thus, it is hypothesized that VEGF-C is indeed involved in tumor cell-tumor microenvironment interactions but, considered alone, without an instrumental role in progressive tumor behavior. The prominent VEGF-C detection in the peritumoral macrovasculature suggests a potential involvement in events ensuring a functional blood supply for tumor survival. In most cases VEGF-C was positive in lymphocytic infiltrates around the tumor glands and in lymphoid follicles at the tumor periphery. These findings indicate that VEGF-C is a factor which appears to be continuously expressed by inflammatory cells in the CC microenvironment so that the inflammatory response might act as a possible source of VEGF-C for paracrine interactions. Considering that immuno-inflammatory reactions play a dual, partly protective, partly potentiating role in tumor progression further studies are needed to confirm the biological relevance of inflammatory cell-associated VEGF-C expression in colorectal cancer [15].

VEGF-D expression was detected particularly in micro- and macrovascular vessels at the invasive front and in the adjacent tumor-free tissue, but showed no association with metastasis. Along with other groups we have also observed a muscularly accentuated and occasionally endothelial VEGF-D expression in the vasculature [16, 17]. It is noteworthy that near the invasive front preexisting large vessels as well as immature, possibly arteriogenetic blood vessels, exhibited VEGF-D. These data indicate that besides its already documented (lymph) angiogenic properties VEGF-D could play a role in the recruitment and possibly also in the arteriogenic process of vessels to maintain a densely branched vascular network in the immediate vicinity of the tumor. In fact, it was reported that a VEGF-D subunit may promote arteriogenesis [18].

In the literature discrepancies exist regarding the clinical significance of VEGF-C and VEGF-D for colorectal cancer metastasis. In accordance with our observations, other research groups also did not establish correlations between tumor cell expression of VEGF-C and lymph node/distant colorectal cancer metastasis [19–22]. In contrast, a number of studies reported that VEGF-C protein expression is significantly related to lymph node metastasis [23–29]. In other published reports, VEGF-C expression, which was detected only at the deepest invasive site of the colon tumor tissue, was significantly correlated with lymphogenous and haematogenous metastasis [30, 31]. While most studies showed a significant correlation between VEGF-D protein expression and nodal metastasis, and in one study even liver metastasis [20, 21, 23, 32, 33], others failed to find any association with lymph node involvement [34]. In all cited studies the immunohistochemical analysis was performed exclusively in carcinoma cells rather than in the tumor-associated vasculature. Generally, the conflicting findings *in situ* concerning the correlation between VEGF-C and VEGF-D protein expression and metastasic behavior of colorectal cancer do not permit a clear assessment. The analysis of both ligands in tumor tissue is complicated by the fact that these are proteolytically processed proteins. VEGF-C and VEGF-D are produced as prepropeptides and are further processed to a biologically fully active form that effectively binds to VEGFR-2 and −3 [12, 13]. These biomolecules can therefore be detected in various forms and subunit compositions with different functions, which should be taken into account in future investigations [35].

Interestingly, there was a close correlation between VEGF-D and its receptor VEGFR-2 expression in both vasculature and cancer cells, suggesting a tumor cell-associated and vessel-related VEGF-D/VEGFR-2 autocrine link in CC, but without direct impact on metastatic spread. This finding is of crucial therapeutic importance because it has been proposed that regarding tumor angiogenesis VEGF-D is an alternative mediator to VEGF-A. This might contribute to mechanisms of resistance to bevacizumab, a widely used anti-cancer drug targeting VEGF-A [36, 37].

In the invasive front in almost all cases capillaries and small vessels were VEGFR-2 positive. It is known that the most aggressive part of the tumor with a high capacity for tumor cell dissociation and initiation of angiogenesis is located in the invasive front [38]. Nowhere else is the topographical contact between tumor cells and functionally active microvasculature so close as at the invasive front. Thus, the dominant presence of VEGFR-2 in the strategically important tumor-host interface compartment supports a pivotal role for this receptor in tumor-vasculature interactions and angiogenesis.

Two observations of vascular VEGFR-2 expression in distant metastatic CC are of importance. In a relatively large number of carcinomas with distant metastasis VEGFR-2 positivity was observed consistently in large vessels in all zones. This could be interpreted as an intimate involvement of VEGFR-2 in the vascularization as well as vessel survival processes, which are especially important for metastatic CC that are particularly characterized by hypoxia-induced, necrosis-rich areas [39]. In the extratumoral soft tissue VEGFR-2 positive capillaries occurred significantly more frequently in distant metastatic CC. It is important to stress that the morphologically “normal appearing” tissue in the tumor vicinity, although tumor-free, is not a physiological tissue. On the contrary, it is an extratumoral area which actively participates in an intricate crosstalk between tumor and neighboring tissue, influencing tumor behavior by interacting protein secretion and receptor activation [40].

In a previous immunohistochemical analysis of almost the same number of colon and rectum carcinomas low tumoral VEGFR-2 expression was associated with lymph node metastasis [41]. In the present study, VEGFR-2 protein exclusively expressed in CC cells, had a uniform staining intensity and distribution in non-metastatic and metastatic cases. We suggest that this discrepancy highlights the distinct nature of these two types of intestinal cancer.

A pVEGFR-2^Tyr1175^ and pVEGFR-2^Tyr1214^ endothelial expression and concomitant expression with VEGFR-2 was demonstrated in all vascular segments in a large number of the carcinomas, suggesting a widespread receptor activation in autoregulatory behavior. The close correlation between VEGFR-2 and pVEGFR-2^Tyr1214^ expression in capillaries and small vessels reveals the receptor autophosphorylation at Tyr1214 as a main event for vascular VEGFR-2-mediated signaling in CC. There were more cases with pVEGFR-2 positivity in endothelial cells than tumors with endothelial VEGFR-2 expression. This finding indicates that ligand-independent receptor phosphorylation is alternatively available for VEGFR-2 activation in CC. Indeed, besides the classical model of VEGFR-2 activation through receptor dimerization and autophosphorylation induced by ligands, the option of ligand-independent phosphorylation through other kinases under cancerous conditions such as vascular fluid shear stress and oxidative stress can also occur [42, 43]. Another ligand-independent VEGFR2 signaling pathway is an endocytic process with internalization of the receptor and its activation in cytosolic regions, such as the Golgi compartment [43, 44]. This intracellular transport in turn results in progressive decrease of VEGFR-2 at the cell surface.

Within the tumor cells, cytoplasmic immunoreactivity accompanied by a simultaneous nuclear expression was seen in almost all carcinomas in the case of pVEGFR-2^Tyr1175^ and in 64 % of the carcinomas regarding pVEGFR-2^Tyr1214^. Both expression profiles were not associated with the metastatic status. A translocation of pVEGFR-2 to the nucleus in neoplastic cells, including colon carcinoma cells, has already been reported [45, 46]. These data suggest that in addition to its signal transduction function VEGFR-2 is involved in the transcriptional processes of gene regulation.

Both phosphorylated receptor forms, but especially pVEGFR-2^Tyr1214^, were frequently expressed in tumor budding regions, where a significant correlation between the receptor and its phosphorylated form Tyr1214 was found. These observations indicate that VEGFR-2 activation by autophosphorylation in dissociated tumor cells could possibly mediate the maintenance of an infiltrative phenotype and the enhancement of migration capacity in colon cancer.

In almost all cases both pVEGFR-2 forms were expressed in tumor-associated inflammatory cells without immunohistochemical detection of the total protein. In accordance with our results, Shin et al. reported that activated T lymphocytes transcribed mRNA for VEGFR-1 and VEGFR-2, but only VEGFR1 was expressed on the T cell surface [47]. Further investigations at both mRNA and protein levels with additional VEGFR-2 antibodies are necessary to clarify this specific observation. Nevertheless, the widespread detection of pVEGFR-2 in inflammatory cells suggests a potential role for the VEGFR-2 activating pathway in the regulation of immune responses in CC.

## Conclusion

Our data indicate that the VEGFR-2 activating pathway with the ligands VEGF-C and VEGF-D and the phosphorylated receptor forms pVEGFR-2^Tyr1175^ and pVEGFR-2^Tyr1214^ is closely involved in events affecting tumor cells themselves as well as components of the tumor microenvironment related to blood supply, angiogenesis and inflammatory response in colon carcinoma. These processes appear to contribute to tumor survival and growth as well as maintenance of the infiltrative phenotype rather than to promote metastasis.
